# The role of MRI and clinicopathologic features in predicting the invasive component of biopsy-confirmed ductal carcinoma in situ

**DOI:** 10.1186/s12880-020-00494-z

**Published:** 2020-08-12

**Authors:** Ga Young Yoon, Woo Jung Choi, Joo Hee Cha, Hee Jung Shin, Eun Young Chae, Hak Hee Kim

**Affiliations:** 1 Department of Radiology, Gangneung Asan Hospital, University of Ulsan College of Medicine, 38 Bangdong-gil, Sacheon-myeon, Gangneung-si, Gangwon-do 25440 Korea; 2 Department of Radiology and Research Institute of Radiology, Asan Medical Center, University of Ulsan College of Medicine, 88 Olympic-ro 43-gil, Songpa-gu, Seoul, 05505 Korea

**Keywords:** Breast, MRI, Ductal carcinoma in situ, Invasive ductal carcinoma

## Abstract

**Background:**

The upgrade rate of biopsy-confirmed ductal carcinoma in situ (DCIS) to invasive carcinoma is up to 50% on final pathology. We investigated MRI and clinicopathologic predictors of the invasive components of DCIS diagnosed by preoperative biopsy and then compared MRI features between patients with DCIS, microinvasive ductal carcinoma (mIDC), and invasive ductal carcinoma (IDC) diagnosed on final pathology.

**Methods:**

Two hundred and one patients with 206 biopsy-confirmed DCIS lesions were enrolled. MRI and clinicopathologic features were used to predict either mIDC or IDC via a cumulative logistic regression analysis. For the lesions detected on MRI, morphologic and kinetic analyses were performed using the Chi-square, Fisher’s exact, and Kruskal-Wallis tests.

**Results:**

Of all the lesions, 112 (54.4%) were diagnosed as DCIS, 50 (24.3%) were upgraded to mIDC, and 44 (21.4%) to IDC. The detection on MRI as mass (Odds ratio (OR) = 8.84, 95% confidence interval (CI) = 1.05–74.04, *P* = 0.045) or non-mass enhancement (NME; OR = 11.17, 95% CI = 1.35–92.36, *P* = 0.025), negative progesterone receptor (PR; OR = 2.40, 95% CI = 1.29–4.44, *P* = 0.006), and high Ki-67 level (OR = 2.42, 95% CI = 1.30–4.50, *P* = 0.005) were significant independent predictors of histologic upgrade. On MRI, 87 (42.2%) lesions appeared as mass and 107 (51.9%) as NME. Irregularly shaped, not-circumscribed, heterogeneous, or rim-enhancing masses with intratumoral high signal intensity or peritumoral edema, clumped or clustered ring-enhancing NMEs, and high peak enhancement were significantly associated with histologic upgrade (*P* < 0.001).

**Conclusion:**

MRI detection, negative PR, and high Ki-67 levels are associated with a histologic upgrade in patients with biopsy-confirmed DCIS. Suspicious MRI features are more frequent in such patients.

## Background

Ductal carcinoma in situ (DCIS) is histologically characterized by the proliferation of malignant epithelial cells within the lumen of the mammary duct, with no evidence of invasion beyond the basement membrane [1]. These cancers are not a single entity but represent a spectrum of diseases [2]. Microinvasive ductal carcinoma (mIDC) is defined as an extension of the cancer cells beyond the basement membrane but not exceeding 1 mm in the greatest dimension, according to the 7th edition of the American Joint Committee on Cancer (AJCC) Cancer Staging Manual [3]. Although DCIS is diagnosed preoperatively by core needle biopsy (CNB) or vacuum-assisted core biopsy, 50% of the biopsy-confirmed DCIS lesions are upgraded to invasive carcinoma when they are surgically excised [4–7]. Treatment plans and prognoses differ between DCIS, mIDC, and invasive ductal carcinoma (IDC), although mIDC is classified as an invasive carcinoma. DCIS and mIDC may be similar in pathology and clinical outcome [8]; however, the differentiation between the two is important to determine whether sentinel lymph node biopsy should be performed [9]. In addition, it is clinically important to recognize the possibility of an underestimation of invasive disease when using a preoperative biopsy to diagnose DCIS [10, 11].

DCIS typically manifests as calcifications visible on mammography [12]; however, ultrasound (US) and magnetic resonance imaging (MRI) play an important role in detecting and assessing the extent of these lesions [13]. Several prior studies have described the US [14, 15] and MRI features [6, 16–20] that can predict the invasive component of a biopsy-proven DCIS. However, there have been few reports that have compared MRI findings between pure DCIS and mIDC cases [21]. To the best of our knowledge, no previous report has compared the predictors of DCIS with those of the more invasive components of the disorder, including mIDC, using cumulative statistical analysis.

The purpose of our current study was to investigate the role of MRI and clinicopathologic features in predicting the invasiveness of preoperative biopsy-confirmed DCIS, and to compare the MRI features among patients with DCIS, mIDC, or IDC diagnosed on final pathology.

## Methods

### Patients

The institutional review board of our hospital approved this study and the requirement for informed consent was waived due to the retrospective nature of the analyses.

**Fig. 1:**
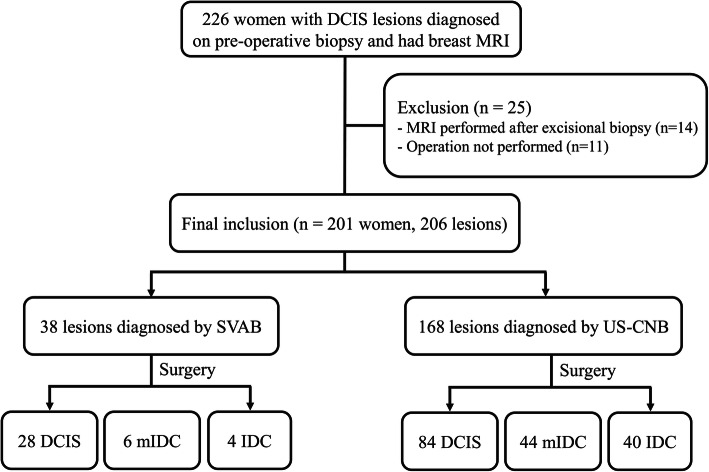
Flow chart for inclusion of patients. DCIS, ductal carcinoma in situ; mIDC, microinvasive ductal carcinoma; IDC, invasive ductal carcinoma; SVAB, stereotactic vacuum-assisted biopsy; US-CNB, ultrasound-guided core needle biopsy

We conducted a retrospective review of the pathologic database of our institution for patients with a histologic diagnosis of DCIS on stereotactic vacuum-assisted biopsy (SVAB) or US-guided CNB (US-CNB) between January and December 2015. We enrolled patients who underwent preoperative breast MRI. The lesions detected on both mammography and US were confirmed by US-CNB. In general, SVAB was performed with either an 8- or 11-gauge vacuum-assisted biopsy needle (Mammotome; Devicor Medical Products, Cincinnati, OH, USA) on 12 samples harvested from each patient. US-CNB was performed with a 14-guage automated biopsy gun (Stericut; TSK Laboratory, Tochigi, Japan) with five samples obtained from each patient. We excluded patients who had undergone excision before the preoperative MRI (*n* = 14) or had not undergone surgery (*n* = 11). Finally, 201 patients (mean age 49.7 ± 10.7 years) with 206 breast lesions were included in the study cohort (Fig. 1). Five patients had a biopsy-confirmed DCIS in bilateral breasts. Ninety-five patients showed overlap with a previous study that compared surgical outcomes of DCIS with and without preoperative MRI [22]. All patients underwent both initial mammography and US prior to the MRI examination. Mammographic densities were recorded in accordance with the categories of breast composition [23], i.e., almost entirely fatty, scattered fibrograndular tissue (fatty), heterogeneously dense, or extremely dense (dense). The presence of calcification was also recorded from the mammographic images.

### MRI acquisition

Bilateral MRI was performed using a 1.5 T or 3.0 T (Avanto; Siemens Medical Solutions, Erlangen, Germany, Skyra; Siemens Medical Solutions, Erlangen, Germany, Ingenia; Philips, Best, The Netherlands) MR scanner and a dedicated 18-channel phased-array breast coil (Siemens Medical Solutions) with the patient in a prone position. The imaging protocol included a T2-weighted short tau inversion recovery turbo spin-echo pulse sequence (repetition time [TR]/echo time [TE], 1300/131; matrix size, 384 × 384; field of view [FOV], 340 × 340 mm^2^; slice thickness, 1.5 mm for the 1.5 T scanner; TR/TE, 1100/131; matrix size, 256 × 416; FOV, 341 × 210 mm^2^; section thickness,1.5 mm for the 3.0 T scanner) and a dynamic contrast material-enhanced fat-saturated axial three-dimensional T1-weighted fast low-angle shot sequence (TR/TE, 5.0/2.4; matrix size, 384 × 384; FOV, 340 × 340 mm^2^; section thickness, 0.9 mm for the 1.5 T scanner; TR/TE, 5.6/2.5; matrix size, 384 × 384; FOV, 360 × 360 mm^2^; section thickness, 0.9 mm for the 3.0 T scanner), consisting of unenhanced and five contrast-enhanced acquisitions. Contrast material (0.2 mL/kg gadoterate meglumine; UNIRAY®; Dongkook Pharmaceutical Co., Ltd., Seoul, Korea) was power-injected (Spectris; Medrad, Pittsburgh, PA, USA) at a flow rate of 1 mL/s, followed by a 20 mL saline flush.

### Image interpretation and computer-aided diagnosis (CAD) data collection

All MR images were reviewed in consensus by two dedicated breast radiologists (G.Y.Y. and W.J.C., with 4 and 10 years of experience in breast imaging, respectively) using the Breast Imaging Reporting and Data System (BI-RADs) [23]. Reviewers were blinded to the clinicopathologic findings. The level of background parenchymal enhancement (BPE; minimal, mild, moderate, or marked), as well as lesion multifocality, multicentricity, and morphologic features were determined. Lesions were classified into mass or non-mass enhancement (NME) types. In patients with multiple lesions, the index lesion, with the largest diameter, was evaluated. Masses were analyzed according to shape, margin, and internal enhancement; the presence of intratumoral high signal intensity (SI) and peritumoral edema was also evaluated on T2-weighted images (T2WI). Intratumoral high SI was visually determined when the SI of the tumor was higher than, or almost the same as, that of water or the vessels, or higher than that of the surrounding normal parenchymal tissue [24]. Peritumoral edema was defined by high SI around the tumor observed on T2WI [25]. NMEs were analyzed according to their distribution (focal, linear, segmental, regional, multiple regions, or diffuse) and internal enhancement. The kinetic features of each tumor were evaluated using a commercially available CAD system (CADstream, version 6.0.1; Confirma, Kirkland, WA, USA). To measure MRI parameters, pre- and post-contrast T1-weighted image series were transferred to a CAD system, which automatically segmented and calculated the tumor diameter (maximal size of the enhancing lesion), angio volume (volume of total enhancing lesion), peak enhancement (highest pixel SI in the first post-contrast series), and delayed enhancement profiles (proportions of persistent, plateau, and washout enhancing components within a tumor).

### Pathologic analysis

All histopathologic information was obtained from the pathology reports of the surgical specimens. We divided the study patients into three groups according to their final surgical pathologic results: DCIS, mIDC, and IDC. When the histological analysis of the surgical specimen showed microinvasive or invasive foci within a tumor which had been preoperatively diagnosed as DCIS, the tumor was defined as a histologically upgraded lesion. Microinvasion was defined according to the 7th edition of the AJCC Cancer Staging Manual, as an extension of the cancer cells beyond the basement membrane into adjacent tissues but not exceeding 1 mm in the greatest dimension [3]. Invasion was also defined in tissues with basement membrane invasion [3]. Patients with multiple microinvasion foci were included in this classification if the size of the largest focus did not exceed 1 mm. Pathological data included nuclear grade, presence of necrosis, pathologic tumor size, the status of the estrogen receptor (ER), progesterone receptor (PR), and human epidermal growth factor receptor 2 (HER2), and Ki-67 proliferation. Pathologic tumor size was recorded as the largest tumor diameter including both DCIS and invasive component. HER2 positivity was defined as a protein overexpression score of 3+ determined by immunohistochemistry or the presence of gene amplification (positive in situ hybridization) [26]. Ki-67 scores ≥20% were considered high.

### Statistical analysis

Imaging findings and clinicopathologic features were compared between the DCIS, mIDC, and IDC groups using the Chi-square test or Fisher’s exact test for categorical variables, and the ANOVA or Kruskal-Wallis test for continuous variables. Independent predictors of microinvasive and invasive carcinomas were analyzed using cumulative logistic regression analysis. Variables with *P* values < 0.1 in univariate analysis were included in the multivariate analysis using a cumulative logistic regression method with backward elimination. The variable, tumor size on MRI, was excluded from the multivariate analysis due to lack of data (non-visible lesions on MRI). For the lesions detected on MRI, a subgroup analysis was performed in accordance with the MR lesion type. Kinetic features were analyzed using the Chi-square test or Fisher’s exact test for categorical variables, and the Kruskal-Wallis test for continuous variables. All statistical analyses were performed with SPSS software version 20.0 (SPSS, Chicago, IL, USA) or SAS version 9.3 (SAS Institute, Cary, NC, USA). A *P* value < 0.05 was considered statistically significant.

## Results

### Predicting the invasive components of DCIS from clinicopathologic and imaging features

Among the 206 biopsy-confirmed DCIS lesions analyzed in this present study, 112 (54.4%) were found to be pure DCIS in the final surgical pathology, 50 (24.3%) were upgraded to mIDC, and 44 (21.4%) were upgraded to IDC. Thirty-eight and 168 patients were diagnosed by SVAB and US-CNB, respectively. The clinicopathologic features of these samples are summarized in Table 1. The SVAB method (*P* = 0.028) and breast-conserving surgery (*P* < 0.001) were significantly more frequent in the DCIS group than in the mIDC and IDC groups. However, the mean age at diagnosis did not significantly differ between the three groups. Histologically, a higher nuclear grade (*P* = 0.001), larger pathologic tumor size of the surgical specimen (*P* < 0.001), negative ER (*P* < 0.001), negative PR (*P* < 0.001), positive HER2 (*P* < 0.001), and high Ki-67 level (*P* < 0.001) were significantly more frequent in the groups with upgraded lesions.

**Table 1:** Clinicopathologic features of the DCIS, mIDC, and IDC lesions in the study cohort

	DCIS (*n* = 112)	mIDC (*n* = 50)	IDC (*n* = 44)	*P* value
Age, years	48.6 ± 10.3	50.3 ± 11.5	52.1 ± 10.8	0.181
Biopsy method				0.028
SVAB	28 (25.0)	6 (12.0)	4 (9.1)	
US-CNB	84 (75.0)	44 (88.0)	40 (90.9)	
Surgery type				< 0.001
Breast conserving	80 (71.4)	29 (58.0)	15 (34.1)	
Mastectomy	32 (28.6)	21 (42.0)	29 (65.9)	
Nuclear grade				0.001
1	15 (13.4)	4 (8.0)	1 (2.3)	
2	88 (78.6)	30 (60.0)	29 (65.9)	
3	9 (8.0)	16 (32.0)	14 (31.8)	
Necrosis				0.073
Present	54 (47.4)	33 (66.0)	27 (61.4)	
Absent	58 (51.8)	17 (34.0)	17 (38.6)	
Pathologic tumor size (cm)	2.0 ± 1.6	3.7 ± 2.3	4.5 ± 2.4	< 0.001
Estrogen receptor				< 0.001
Positive	103 (92.0)	26 (52.0)	34 (77.3)	
Negative	9 (8.0)	24 (48.0)	10 (22.7)	
Progesterone receptor				< 0.001
Positive	95 (84.8)	21 (42.0)	26 (59.1)	
Negative	17 (15.2)	29 (58.0)	18 (40.9)	
HER2				< 0.001
Positive	20 (17.9)	30 (60.0)	16 (36.4)	
Negative	92 (82.1)	20 (40.0)	28 (63.6)	
Ki-67				< 0.001
High (≥20%)	24 (21.4)	24 (48.0)	24 (54.5)	
Low (< 20%)	88 (78.6)	26 (52.0)	20 (45.5)	

Data values indicate the number of patients (with percentages in parentheses), or the mean ± standard deviation.

*DCIS* ductal carcinoma in situ; *mIDC* microinvasive ductal carcinoma; *IDC* invasive ductal carcinoma; *SVAB* stereotactic vacuum-assisted biopsy; *US-CNB* US-guided core needle biopsy; *HER2* human epidermal growth factor receptor 2

Table 2 presents the imaging features of the DCIS, mIDC, and IDC groups. The presence of calcification on mammography was more frequent in the groups with upgraded lesions (*P* = 0.025). The mIDC and IDC groups tended to show more NME lesions than the DCIS group; however, this difference did not reach statistical significance (*P* = 0.053). There were no significant differences between the groups with respect to parenchymal density on mammography or BPE on MRI.

**Table 2:** Imaging features of DCIS, mIDC, and IDC

	DCIS (*n* = 112)	mIDC (*n* = 50)	IDC (*n* = 44)	*P* value
Mammography				
Parenchymal density				0.492
Fatty	20 (17.9)	8 (16.0)	11 (25.0)	
Dense	92 (82.1)	42 (84.0)	33 (75.0)	
Calcification				0.025
Present	65 (58.0)	40 (80.0)	29 (65.9)	
Absent	47 (42.0)	10 (20.0)	15 (34.1)	
MRI				
BPE				0.145
Minimal or mild	78 (69.6)	39 (78.0)	37 (84.1)	
Moderate or marked	34 (30.4)	11 (22.0)	7 (15.9)	
Multifocality				> 0.999
Yes	5 (4.5)	2 (4.0)	1 (2.3)	
No	107 (95.5)	48 (96.0)	43 (97.7)	
Multicentricity				0.470
Yes	7 (6.3)	1 (2.0)	1 (2.3)	
No	105 (93.8)	49 (98.0)	43 (97.7)	
Type of lesion				0.053
Non-visualization	11 (9.8)	1 (2.0)	0 (0)	
Mass	50 (44.6)	18 (36.0)	19 (43.2)	
NME	51 (45.5)	31 (62.0)	25 (56.8)	

Data values indicate the number of patients (with percentages in parentheses), the mean ± standard deviation, or the median with range.

*DCIS* ductal carcinoma in situ; *mIDC* microinvasive ductal carcinoma; *IDC* invasive ductal carcinoma; *BPE* background parenchymal enhancement; *NME* non-mass enhancement

Among all variables, the followings that showed *P* values < 0.1 on univariate analysis were used as input variables in subsequent multivariate analysis: older age (*P* = 0.070), US-CNB (*P* = 0.009), nuclear grade 3 (*P* = 0.001), presence of necrosis (*P* = 0.044), negative ER (*P* < 0.001), negative PR (*P* < 0.001), positive HER2 (*P* < 0.001), high Ki-67 level (*P* < 0.001), presence of mammographic calcification (*P* = 0.072), minimal or mild BPE (*P* = 0.051), and mass (*P* = 0.041) or NME (*P* = 0.019) detection on MRI. The multivariate analysis revealed that a mass lesion detected on MRI (Odds ratio (OR) = 8.84, 95% confidence interval (CI) = 1.05–74.04, *P* = 0.045), NME lesion detected on MRI (OR = 11.17, 95% CI = 1.35–92.36, *P* = 0.025), negative PR (OR = 2.40, 95% CI = 1.29–4.44, *P* = 0.006), and higher Ki-67 level (OR = 2.42, 95% CI = 1.30–4.50, *P* = 0.005) remained significant independent factors associated with histologic upgrade (Table 3).

**Table 3:** Univariate and multivariate analysis using a cumulative logistic regression method to predict histologic upgrade to microinvasion and invasion

	Univariate analysis		Multivariate analysis	
	Odds ratio (95% CI)	*P* value	Adjusted Odds ratio (95% CI)	*P* value
Age (old vs. young)	1.02 (1.00–1.05)	0.070		
Biopsy method (US-CNB vs. SVAB)	2.78 (1.28–6.01)	0.009		
Nuclear grade (grade 3 vs. grade 1–2)	7.51 (2.38–23.75)	< 0.001		
Necrosis (present vs. absent)	1.74 (1.02–2.98)	0.044		
ER (absent vs. present)	2.97 (1.63–5.38)	< 0.001		
PR (absent vs. present)	3.53 (2.02–6.17)	< 0.001	2.40 (1.29–4.44)	0.006
HER2 (present vs. absent)	2.81 (1.62–4.87)	< 0.001		
Ki-67 (high vs. low)	3.44 (1.98–6.00)	< 0.001	2.42 (1.30–4.50)	0.005
Mammographic calcification (present vs. absent)	1.70 (0.96–3.02)	0.072		
BPE on MRI (minimal or mild vs. moderate or marked)	1.88 (1.00–3.55)	0.051		
MR type of lesion (mass or NME vs. non-visualization)				
Mass	8.81 (1.10–70.93)	0.041	8.84 (1.05–74.04)	0.045
NME	11.95 (1.50–95.20)	0.019	11.17 (1.35–92.36)	0.025

*CI* confidence interval; *US-CNB* US-guided core needle biopsy; *SVAB* stereotactic vacuum-assisted biopsy; *ER* estrogen receptor; *PR* progesterone receptor; *HER2* human epidermal growth factor receptor 2; *BPE* background parenchymal enhancement; *NME* non-mass enhancement

### MRI features in DCIS, mIDC and IDC

**Fig. 2:**
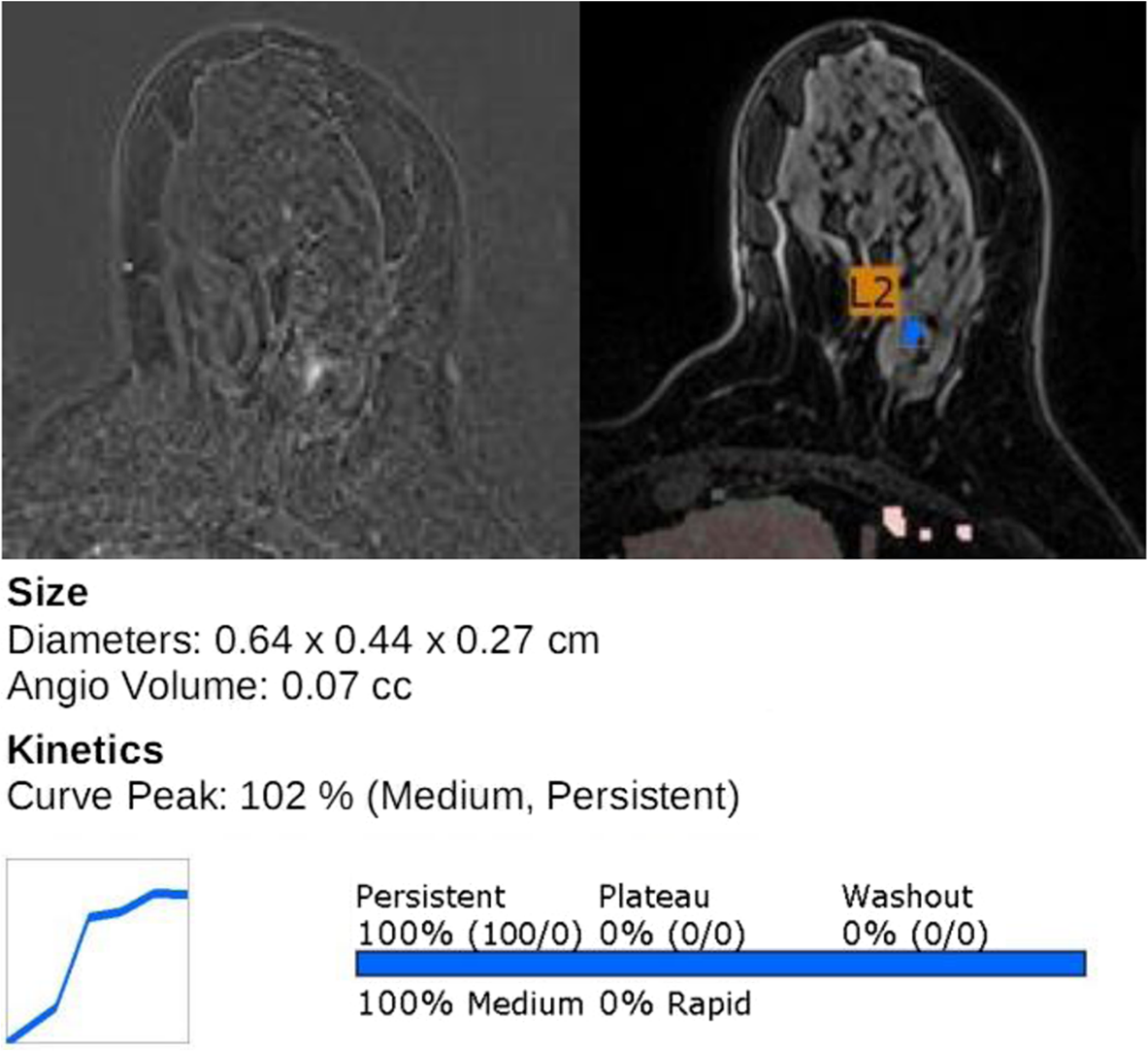
Imaging features of DCIS on final surgical pathology. Axial fat-suppressed T1-weighted contrast-enhanced MRI revealed a small oval-shaped mass with homogeneous enhancement (left). MRI with a CAD color overlay map revealed the tumor size and enhancement kinetics, i.e., a 102% peak enhancement and a 100% persistent component (right)

**Fig. 3:**
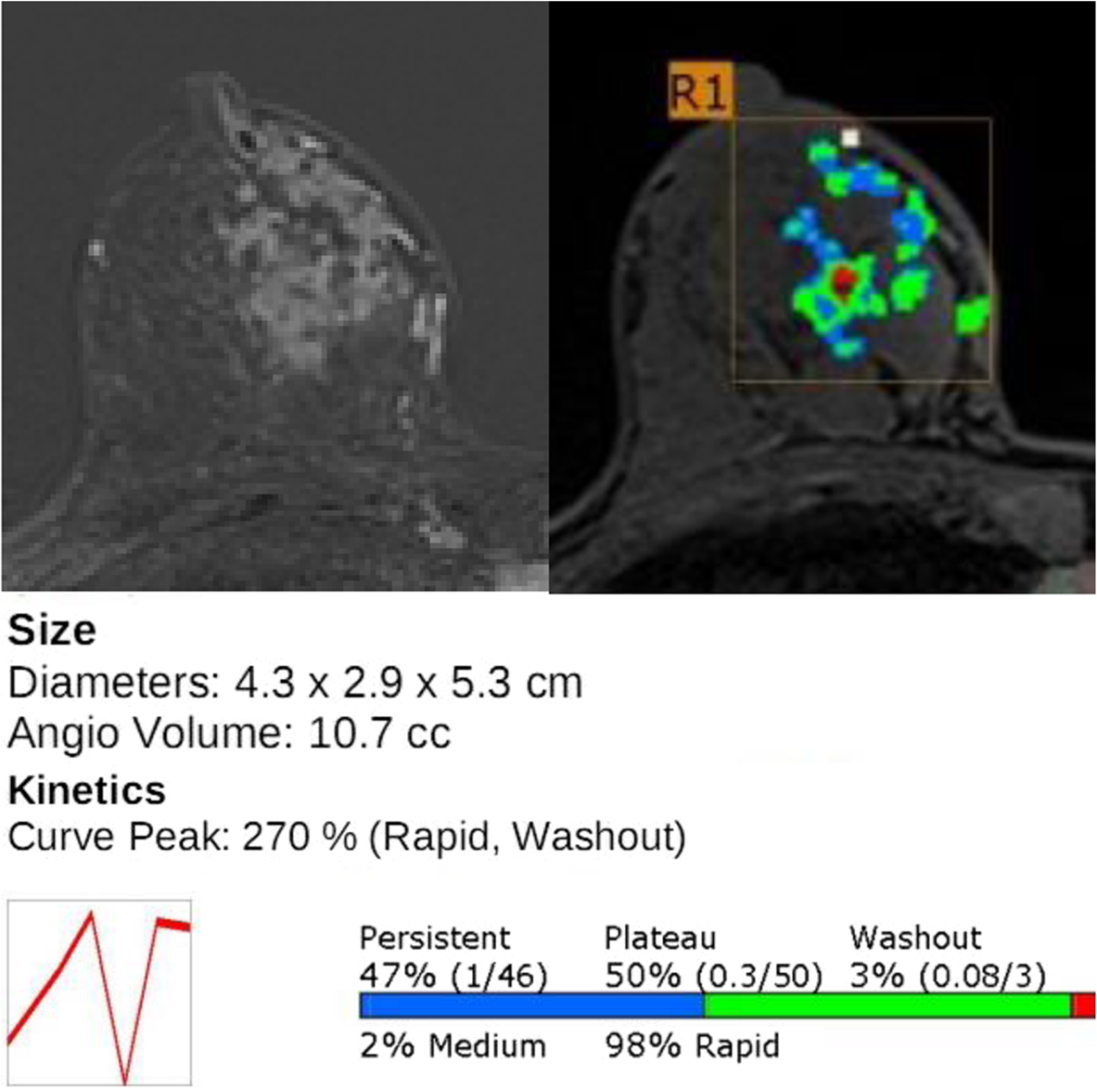
Imaging features of invasive ductal carcinoma on final surgical pathology. Axial fat-suppressed T1-weighted contrast-enhanced MRI showed a segmental distributed non-mass enhancement with clustered ring enhancement (left). MRI with a CAD color overlay map indicated the tumor size and enhancement kinetics, i.e., a 270% peak enhancement and a 3% washout component (right)

Table 4 lists the MRI features by lesion type in the DCIS, mIDC, and IDC groups. In the two invasive groups, the median tumor size for both mass and NME lesions was significantly greater than that in the DCIS group. The dominant imaging features of the mass lesions in the two invasive disease groups were irregular shape and not-circumscribed appearance with heterogeneous or rim enhancement (*P* = 0.001). Conversely, the DCIS lesions showed benign favored features more frequently (Fig. 2). Intratumoral high SI and peritumoral edema on T2WI were also more frequent in the invasive groups than in the DCIS group (*P* < 0.001). NME lesions with clumped or clustered ring enhancement were more frequent in the invasive disease groups than in the DCIS group (*P* = 0.001; Fig. 3). Segmental distribution of NME lesions was common in all three groups, but the invasive groups showed a higher percentage of segmental distribution than the pure DCIS group; however, this difference did not reach statistical significance (*P* = 0.075). Lesions with high peak enhancement percentages were more frequent in mIDC and IDC groups (*P <* 0.001). Other kinetic features of mass and NME lesions, including initial and delayed enhancement, persistent component, plateau component, and washout component did not significantly differ between the three groups.

**Table 4:** MRI characteristics of the DCIS, mIDC, and IDC group

	DCIS (*n* = 101)	mIDC (*n* = 49)	IDC (*n* = 44)	*P* value
Mass (*n* = 87)	DCIS (*n* = 50)	mIDC (*n* = 18)	IDC (*n* = 19)	
MR-measured tumor size (cm)	1.1 [0.9–1.6]	2.2 [0.9–3.3]	2.2 [1.6–5.1]	< 0.001
Shape				0.001
Oval or round	28 (56.0)	4 (22.2)	2 (10.5)	
Irregular	22 (44.0)	14 (77.8)	17 (89.5)	
Margin				0.001
Circumscribed	21 (42.0)	3 (16.7)	0 (0.0)	
Not-circumscribed	29 (58.0)	15 (83.3)	19 (100.0)	
Enhancement pattern				0.001
Homogeneous	19 (38.0)	2 (11.1)	0 (0.0)	
Heterogeneous	26 (52.0)	12 (66.7)	11 (57.9)	
Rim	5 (10.0)	4 (22.2)	8 (42.1)	
Intratumoral high SI on T2WI	6 (12.2)	4 (22.2)	11 (61.1)	< 0.001
Peritumoral edema on T2WI	0 (0.0)	3 (16.7)	6 (33.3)	< 0.001
NME (*n* = 107)	DCIS (*n* = 51)	mIDC (*n* = 31)	IDC (*n* = 25)	
MR-measured tumor size (cm)	3.8 [2.3–5.0]	4.9 [3.1–6.3]	5.8 [4.2–6.6]	0.002
Distribution				0.075
Segmental	34 (66.7)	26 (83.9)	19 (76.0)	
Linear	2 (3.9)	0 (0.0)	0 (0.0)	
Focal	10 (19.6)	3 (9.7)	2 (8.0)	
Regional	4 (7.8)	2 (6.5)	0 (0.0)	
Diffuse	1 (2.0)	0 (0.0)	4 (16.0)	
Enhancement pattern				0.001
Homogeneous	17 (33.3)	0 (0.0)	3 (12.0)	
Heterogeneous	16 (31.4)	5 (16.1)	4 (16.0)	
Clumped	14 (27.5)	22 (71.0)	14 (56.0)	
Clustered ring	4 (7.8)	4 (12.9)	4 (16.0)	
Kinetic feature (*n* = 194)				0.763
Initial enhancement				
Slow	2 (2.0)	2 (4.1)	2 (4.5)	
Medium	5 (5.0)	1 (2.0)	2 (4.5)	
Fast	94 (93.1)	46 (93.9)	40 (90.9)	
Delayed enhancement				0.383
Persistent	13 (12.9)	5 (10.2)	4 (9.1)	
Plateau	16 (15.8)	4 (8.2)	3 (6.8)	
Washout	72 (71.3)	40 (81.6)	37 (84.1)	
Peak enhancement (%)	171.0 [127.5–220.0]	206.0 [153.0–282.5]	230.0 [190.5–294.0]	< 0.001
Persistent component (%)	60.0 [28.0-, 84.0]	70.0 [49.5, 86.5]	54.5 [36.0–76.0]	0.205
Plateau component (%)	28.0 [12.5–43.0]	24.0 [10.5–37.0]	30.0 [19.0–40.5]	0.343
Washout component (%)	5.0 [0.1–16.0]	2.0 [0.3–10.0]	7.5 [1.3–19.8]	0.254

Data indicate the number of lesions (with percentages in parentheses) or the median values with range.

*DCIS* ductal carcinoma in situ; *mIDC* microinvasive ductal carcinoma; *IDC* invasive ductal carcinoma; *SI* signal intensity; *T2WI* T2-weighted image; *NME* nonmass enhancement

## Discussion

We found in the present analysis that approximately 45.6% of lesions with a preoperative diagnosis of DCIS experience a postoperative histopathologic upgrade. Our current findings demonstrated that detectability on MRI, PR negativity, and a high Ki-67 level were significant independent factors associated with a histologic upgrade from DCIS. With regard to MRI features of DCIS lesions, an irregular shape, not-circumscribed margins, heterogeneous or rim-enhancing masses, clumped or clustered ring-enhancing NMEs, a larger size, and high peak enhancement were significantly associated with histologic upgrade. In these cases, histologic upgrade should be considered before surgical planning, to include sentinel lymph node biopsy in view of possible axillary lymph node metastasis.

In this study, histologic upgrade rates were consistent with those of previous studies, although the proportion was relatively high [4–7]. This might be related to the method of biopsy. We performed US-guided CNB more frequently than SVAB. Previous studies have reported that greater sample numbers and larger tumor volumes documented by SVAB are associated with a lower rate of histologic upgrade [6, 7, 20].

The presence of a lesion on MRI was found to be a significant independent factor associated with histologic upgrade. A multivariate analysis showed the highest OR for the NME lesions (OR = 11.17), followed by mass lesions (OR = 8.84). Many prior studies have reported that the presence of a mass lesion on an MR image is a preoperative predictor of DCIS with an invasive component [6, 7, 20, 27, 28]. Our current results are not consistent with these previous findings; however, this discrepancy might be related to the lesion sizes in our present study series. Among the lesions detected on MRI in our current study, a larger MR-measured tumor size was dominant in both mass and NME types in the histologic upgrade groups, with NME lesions found to be larger than mass lesions. A relatively large lesion on MRI may be associated with invasive disease. Consistent with our present findings, previous studies have reported that tumor size is a predictor of DCIS with invasive components [20, 27, 29]. Among the 12 non-visualized lesions on MRI in our current study series, 11 were confirmed to be DCIS and one, mIDC.

In terms of the MRI morphologic features of diagnosed DCIS, an irregular shape, not-circumscribed margins, rim-enhancing masses, and clumped or clustered ring-enhancing NMEs were significantly associated with a histologic upgrade in our study. This is consistent with previous reports indicating that suspicious morphologic imaging features indicate a likelihood of malignancy [16, 20, 30–34]. Hahn et al. reported that a spiculated mass, segmental distribution, clustered ring enhancement of an NME, and enhancement kinetics showing a strong initial enhancement with subsequent washout were significantly more frequent in mIDC than in DCIS [16]. Tozaki et al. demonstrated that clustered ring enhancements had the highest positive predictive value for malignancy in NME lesions [32, 33]. In our current analyses, we found no significant differences in NME distribution between the three groups; however, the invasive groups showed higher percentages of segmental distribution than the pure DCIS group. This finding is consistent with that of previous studies [16, 29], and although this difference did not reach statistical significance in our analyses, segmental distribution of NMEs might be helpful in the diagnosis of invasive disease.

Among the mass lesions detected in our present cohort, intratumoral high SI, and peritumoral edema on T2WI were more frequent in the histologic upgrade groups than in the pure DCIS group. Previous studies reported that aggressively invasive breast cancers rapidly outgrow their blood supply, leading to areas of hypoxia within the tumor and subsequently to necrosis [24, 35]. Thus, these reports suggest that intratumoral high SI on T2WI, which is suggestive of intratumoral necrosis, is a prognostic indicator of invasive breast cancer. Several other studies found that peritumoral edema on T2WI is a clue to the diagnosis of invasive cancer [25, 29]. Our present results are consistent with the findings of these previous reports, and suggest that these imaging findings may be helpful in differentiating histologic upgrade lesions from pure DCIS lesions.

Based on the MRI kinetic features of the lesions analyzed in our present study, a higher peak enhancement was indicative of more invasiveness. Among the kinetic parameters, peak enhancement is known to reflect contrast concentrations both in intra- and extravascular interstitial spaces [36]. Nam et al. have reported that higher peak enhancement is associated with poorer disease-free survival rates and greater tumor aggressiveness [37]. We speculate that these prior results might explain the relationship between a peak enhancement and histologic upgrade of a DCIS.

In our current investigation, microcalcification on mammography was more frequent in upgraded mIDC and IDC lesions, although this difference did not reach statistical significance in the multivariate analysis. Among the calcified lesions observed in our series, 28.4% (38/134) were assessed with SVAB, and lesions confirmed by SVAB were more frequent in the pure DCIS group than in the histologic upgrade groups. Several previous studies reported that greater sample numbers and larger tumor volumes documented by SVAB are associated with a lower rate of histologic upgrade [6, 7, 20]. These findings may explain the fact that the microcalcifications seen on both mammography and US may show more frequent upgrade than that seen on mammography alone due to the different imaging findings and sample sizes.

ER and PR negativity as well as HER2 positivity were more frequent in mIDC and IDC lesions in our current series, and a negative PR result was a significant independent predictor of a histologic upgrade from a pure DCIS. Ozkan-Gurdal et al. reported that hormone receptor negativity indicates a higher likelihood of a microinvasive component in DCIS [38]. Wan et al. demonstrated that ER and PR expressions were significantly higher in DCIS than in DCIS with microinvasion [39]. Mylona et al. reported that HER2 overexpression in DCIS might be related to the transformation from carcinoma in situ to invasive cancer [40]. These findings collectively indicate that subsets of DCIS and histologically upgraded lesions may differ at a molecular level.

Ki-67 is a nuclear protein associated with cellular proliferation [41]. Inwald et al. reported that a high Ki-67-labeling index is associated with poorer disease-free survival and overall survival outcomes in breast cancer patients [42]. Our present results demonstrated that high Ki-67 expression is an independent factor associated with a histologic upgrade from DCIS and suggest the potential use of this parameter for predicting an invasive component in patients with biopsy-proven DCIS.

This study had several limitations that should be noted. First, a retrospective design was used and the number of cases was relatively small. Second, two different breast MRI protocols (1.5 T vs. 3 T) were used at our hospital during the study period. Moreover, we did not analyze diffusion weighted imaging findings which might have provided additional information. Third, we included SVAB cases, which made it difficult to interpret the MRI findings. Fourth, our analysis was conducted at a single tertiary referral center and a large, multi-institutional study will be needed to validate the results.

## Conclusions

Detection on MRI, PR negativity, and a high Ki-67 index are significantly associated with a histologic upgrade from biopsy-confirmed DCIS to mIDC or IDC. In addition, suspicious MRI features are more frequent in such histologic upgrade groups. Our present results may be helpful for predicting invasiveness in DCIS cases and thus devising a more appropriate surgical approach.

## Data Availability

All data analyzed during this study are included in this published article. The datasets analysed during the current study available from the corresponding author on reasonable request.

## Abbreviations

DCIS: Ductal carcinoma in situ
mIDC: Microinvasive ductal carcinoma
IDC: Invasive ductal carcinoma
US: Ultrasound
MRI: Magnetic resonance imaging
CNB: Core needle biopsy
SVAB: Stereotactic vacuum-assisted biopsy
BPE: Background parenchymal enhancement
NME: Non-mass enhancement
T2WI: T2-weighted images
SI: Signal intensity
CAD: Computer-aided diagnosis
ER: Estrogen receptor
PR: Progesterone receptor
HER2: Human epidermal growth factor receptor 2
OR: Odds ratio
